# Identification of Pancreatic Neuroendocrine Tumor During Evaluation for Severe Valvulopathy in a Patient With a History of Lung Carcinoid Tumor: A Case Report

**DOI:** 10.7759/cureus.98742

**Published:** 2025-12-08

**Authors:** Zhan Rong, Cassie Liu, Christopher Loh, Elmira Mostafidi, Zhiheng Pei, Peter Avvento, Viraj Modi

**Affiliations:** 1 Internal Medicine, Stony Brook University Hospital, Stony Brook, USA; 2 Pathology, State University of New York (SUNY) Downstate Medical Center, Brooklyn, USA; 3 Pathology, New York University Grossman School of Medicine, New York, USA; 4 Internal Medicine, Northport VA Medical Center, Northport, USA

**Keywords:** carcinoid tumor, heart failure, metastatic malignancy, pancreatic neuroendocrine tumor, second primary neoplasm

## Abstract

A 36-year-old man with a history of resected typical lung carcinoid tumor and bicuspid aortic valve presented with acute decompensated heart failure. Workup revealed severe mitral valvulopathy and incidentally identified an enhancing pancreatic head mass with additional lesions on imaging. Liver biopsy was benign, while endoscopic ultrasound-guided biopsy of the pancreatic mass showed a well-differentiated, WHO grade 1 pancreatic neuroendocrine tumor (NET) positive for chromogranin, synaptophysin, and CD56, with Ki-67 < 3%. Serum 5-hydroxyindoleacetic acid (5-HIAA) and chromogranin A were elevated. Positron emission tomography (PET)-DOTATATE demonstrated somatostatin receptor-avid lesions in the pancreas, liver, bone, and lymph nodes, consistent with metastatic neuroendocrine neoplasm. The patient underwent aortic valve replacement followed by long-acting octreotide therapy. Concurrent duodenal biopsies revealed celiac disease, and his gastrointestinal symptoms improved with a gluten-free diet. This case represents an exceptionally rare scenario of pancreatic neuroendocrine tumor in a patient with prior lung carcinoid tumor and raises the critical question of pancreatic metastasis versus a second primary NET in the absence of hereditary syndromes. It underscores the need for heightened vigilance for second primary malignancies (SPMs) and atypical metastatic patterns in patients with a history of NETs and highlights the importance of multidisciplinary evaluation for accurate classification and optimal management.

## Introduction

Neuroendocrine tumor (NET) is a broad umbrella term that defines neoplasms that arise from neuroendocrine cells regardless of the organ of origin [1]. Among the organs that NETs can originate from, the lung and pancreas are two of the well-known ones.

Lung NETs include small cell lung carcinomas (SCLC), large cell neuroendocrine carcinomas, and typical and atypical carcinoid tumors. Among these different types of lung NETs, lung carcinoid, including both typical and atypical types, is extremely rare, comprising 1%-2% of all lung cancers [2]. About half of lung carcinoid cases are diagnosed incidentally [3] in asymptomatic patients. Carcinoid syndrome, with the presentation of flushing, diarrhea, wheezing, and right-sided heart disease, is present in only 2%-12% of patients diagnosed with lung carcinoid [3]. Metastasis is frequently seen in patients with lung carcinoid and occurs in up to 70% of patients diagnosed with atypical lung carcinoid tumors [3].

Other than being from lung origin, NETs can also be of pancreatic origin. Pancreatic NET comprises 1%-2% of all pancreatic malignancies [4] and has an incidence of <1 case per 100,000 people [5]. When pancreatic NETs secrete hormones, they are categorized as functional [5]. Some of the most common hormones secreted by pancreatic NETs include insulin, glucagon, gastrin, and vasoactive intestinal polypeptide (VIP) [5]. Symptoms caused by these neuroendocrine tumors are largely dependent on the hormones they secrete. Pancreatic NETs can also secrete serotonin (5-HT) on rare occasions [6]. However, the incidence of 5-HT-secreting pancreatic NET is unclear.

Given the rarity of lung carcinoid and pancreatic NETs, it is not hard to imagine that lung carcinoid with pancreatic metastasis is exceedingly rare. Lung carcinoid is known to metastasize to sites such as intrathoracic lymph nodes, liver, and central nervous system [4]. In comparison, there is very little literature that reports pancreatic metastasis, including only a few case reports [4,7]. Even when considering the broader category of all primary lung neuroendocrine tumors, metastasis to the pancreas only occurs in 0.7% of cases [8]. Therefore, there is no standardized guideline on screening for pancreatic metastasis in patients with a history of lung carcinoid, although some patients may present with symptoms suspicious for NETs. In addition, the incidence of two primary NETs in the same patient is also exceedingly rare among those without inherited oncologic syndromes such as multiple endocrine neoplasia (MEN). However, second primary malignancy (SPM) is much more common in patients with a history of NET compared to the general population, with an incidence of 25% [9]. Therefore, active surveillance of SPM may have a beneficial effect in early detection and intervention in patients with NETs. In this case report, we present a patient with a history of lung carcinoid in remission who presented with primarily cardiac symptoms and was subsequently found to have a pancreatic NET, highlighting the challenges in diagnosing rare NETs and calling to attention the need for physicians to be vigilant about SPMs, even second NETs in patients with a history of NETs.

## Case presentation

**Figure 1:**
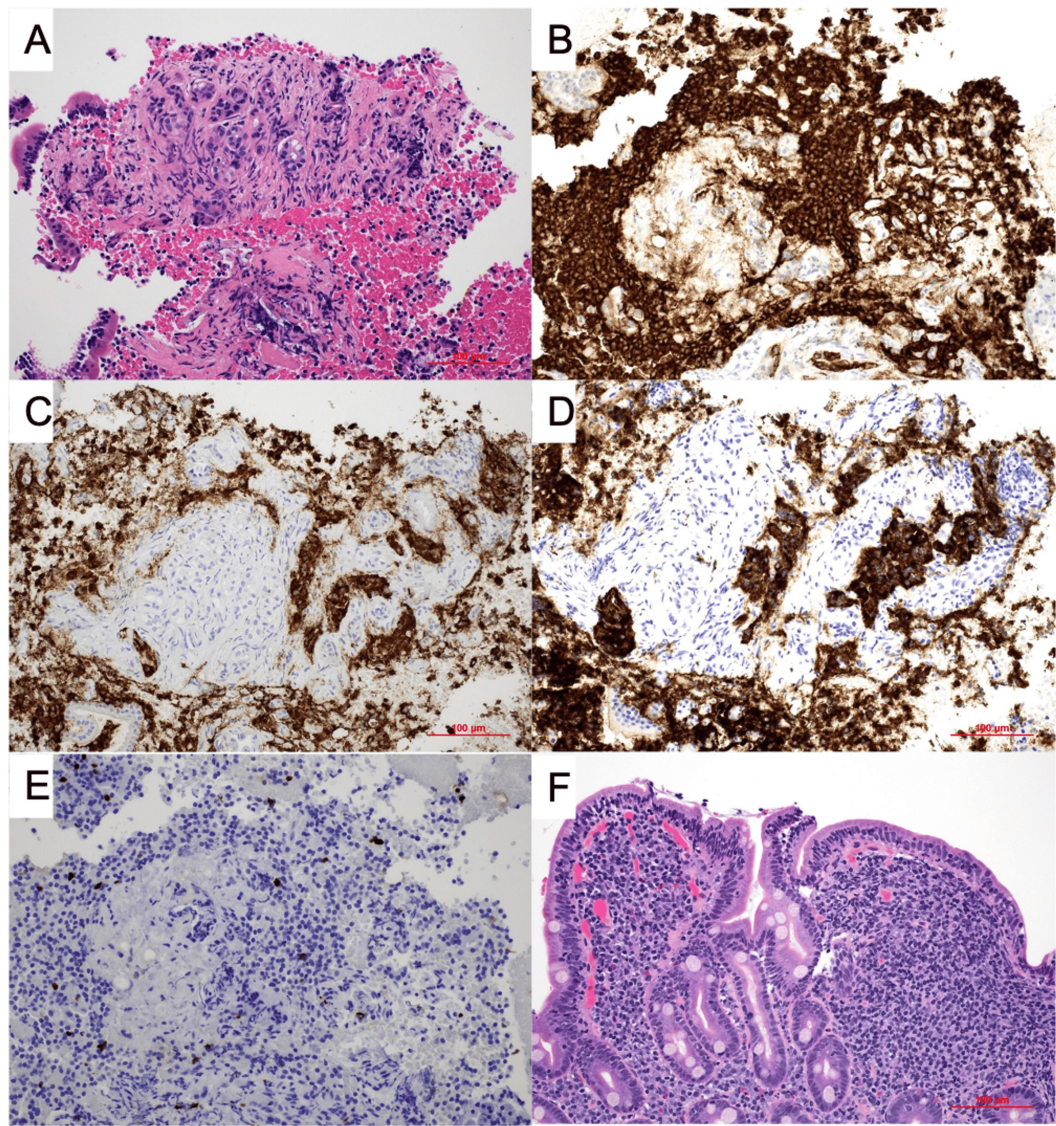
Pathology slides of pancreatic NET and duodenal biopsy (A) H&E stain (200×) showing nested proliferation of bland monomorphic cells with round to oval nuclei, fine granular chromatin, and moderate eosinophilic granular cytoplasm. No necrosis or mitotic figures were identified. (B) CD56 immunohistochemistry (200×) highlights tumoral cells with strong and diffuse membranous positivity. (C) Chromogranin immunohistochemistry (200×) highlights the majority of tumoral cells with strong cytoplasmic positivity. (D) Synaptophysin immunohistochemistry (200×) highlights the majority of tumoral cells with strong cytoplasmic positivity. (E) Ki67 immunohistochemistry (200×) shows a low proliferative index (less than 3%). (F) H&E stain (200×) shows duodenal mucosa with partial villous atrophy, increased intraepithelial lymphocytes, and crypt hyperplasia, consistent with celiac disease. NET: neuroendocrine tumor, H&E: hematoxylin and eosin

A 36-year-old man with a lung carcinoid tumor treated with left lower lobe lobectomy at age 23, bipolar disorder on lithium, and a bicuspid aortic valve presented to the Northport VA Medical Center with two weeks of worsening intermittent dyspnea, orthopnea, nausea, and vomiting. On admission, the patient’s vital signs were stable. His physical examination showed bilateral lower extremity edema. Laboratory work was significant for an elevated brain natriuretic peptide (BNP) up to 1,143 pg/mL, normal troponin, normal thyroid-stimulating hormone (TSH), and normal liver function tests. Electrocardiogram (EKG) showed a new left bundle branch block (LBBB). His echocardiogram showed a newly reduced ejection fraction (EF) of <20% with severe left ventricular dilatation and severe mitral regurgitation (prior EF: 52%-73%), concerning for new-onset acute decompensated heart failure (ADHF). The patient was treated with diuresis and started on guideline-directed medical therapy (GDMT). Given facility availability, he was later transferred to the Manhattan VA for continued cardiac workup. Right heart catheterization showed no evidence of ischemic cardiomyopathy. During initial imaging with computed tomography (CT) of the thorax, he was incidentally found to have an enhancing pancreatic head mass with liver lesions. He underwent a biopsy of liver lesions showing normal liver parenchyma. Biopsy of the pancreatic head mass then showed cells that stained positive for chromogranin, synaptophysin, and CD56, and negative for AE1/AR3, consistent with a well-differentiated neuroendocrine tumor, WHO grade 1 (Figure 1).

**Figure 2:**
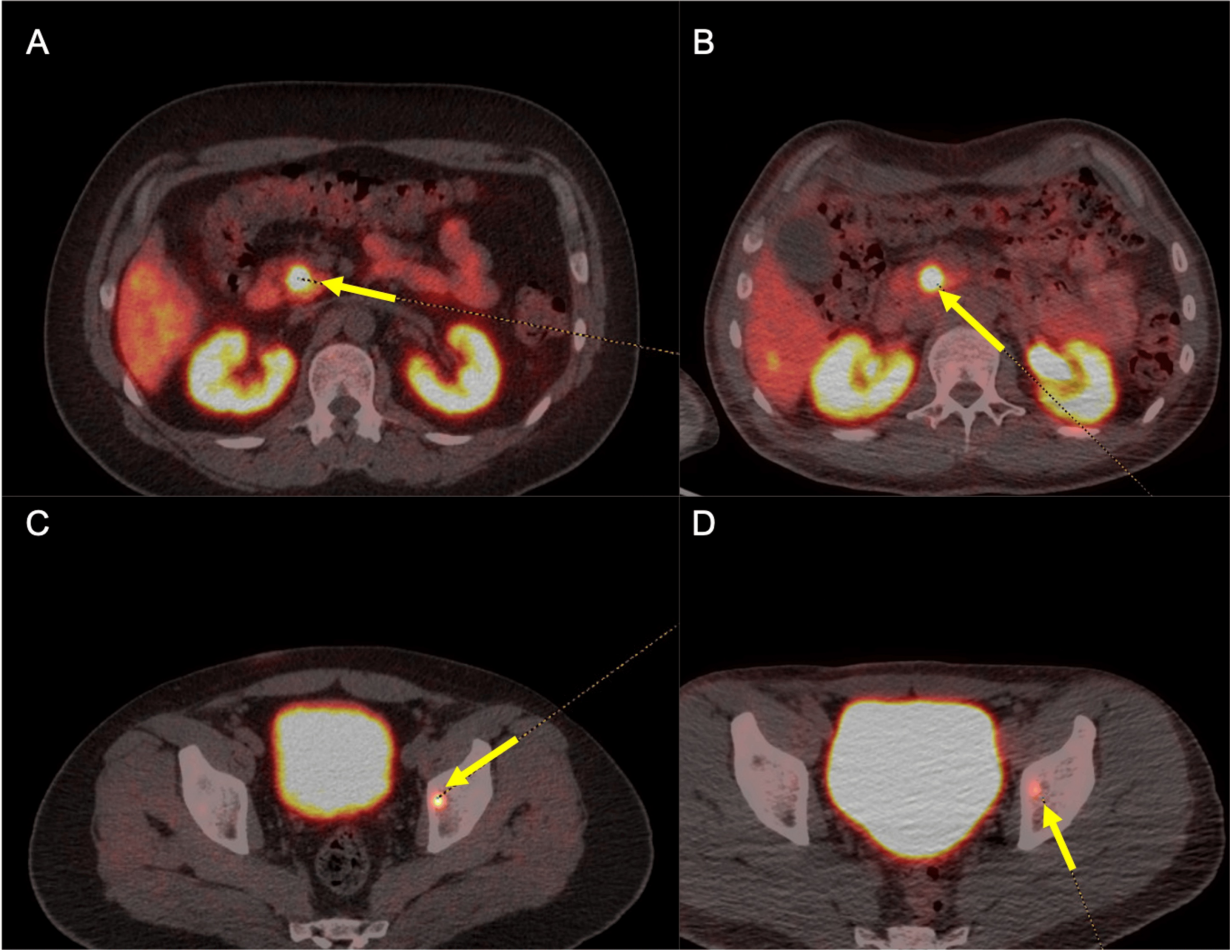
PET-DOTATATE images of active lesions before and after treatment (A) Pancreatic head lesion before octreotide treatment. (B) Pancreatic head lesion after three doses of octreotide, showing mild response. (C) Left iliac lesion before octreotide treatment. (D) Left iliac lesion after three doses of octreotide treatment, showing moderate response. PET: positron emission tomography

Ki67 proliferative index was less than 3%. During his hospitalization, serum 5-hydroxyindoleacetic acid (5-HIAA) level was elevated at 22 mg/L, 24-hour urine 5-HIAA level was normal, and chromogranin A was mildly elevated at 126 ng/mL. Interestingly, his duodenal biopsy showed celiac disease. Cardiac magnetic resonance imaging (MRI) showed a bicuspid aortic valve with moderate to severe regurgitation. He was stable after acute treatment and discharged with oncology follow-up. His outpatient positron emission tomography (PET)-DOTATATE scan showed abnormal tracer avid signals at the pancreatic head, body of pancreas, left cardiac apex, T2 vertebrae and left inguinal lymph nodes, liver foci, hepatoportal lymph nodes, and midline posterior falx, consistent with metastatic disease. At the time of manuscript submission, the patient had undergone aortic valve replacement before receiving treatment with octreotide 30 mg IV monthly for three doses, with a plan for a post-treatment PET-DOTATATE scan (Figure 2).

His nausea improved after adhering to a gluten-free diet. Pathology from his replaced aortic valve showed congenital bicuspid morphology with associated chronic degenerative changes and focal Congo red-positive amyloid deposits within areas of fibrosis, which were localized and not indicative of systemic amyloidosis. Notably, there was no histologic evidence of carcinoid heart disease with no plaque-like endocardial or valvular deposits characteristic of serotonin-induced injury.

## Discussion

This case illustrates the exceptional rarity of a patient with a history of lung carcinoid tumor subsequently presenting with a pancreatic neuroendocrine tumor (NET). While lung carcinoids represent only 1%-2% of all lung cancers, their metastasis to the pancreas is exceedingly rare, with an incidence of only 0.7% among all lung NETs [3,8]. The literature documents only a handful of case reports describing such occurrences, underscoring the absence of standardized guidelines for pancreatic surveillance in patients with prior lung carcinoid [4,7].

In general, patients with NETs face a significantly higher risk of developing SPMs, with an incidence estimated at 25% [9]. This risk is particularly elevated in foregut-derived NETs, suggesting that shared tumorigenic pathways may predispose these patients to subsequent cancers [9]. Although most SPMs in patients with NETs are non-NET cancers, our case raises the possibility of two distinct NET primaries, a phenomenon that is exceedingly rare in the absence of hereditary syndromes such as multiple endocrine neoplasia. Reports of two distinct primary NETs in the same patient without an inherited syndrome are exceedingly rare.

Epidemiological data further demonstrate that the most common sites of SPMs include the gastrointestinal and genitourinary tracts, which aligns with the pancreatic localization observed in our patient [10]. Furthermore, the occurrence of SPMs in patients with NETs has been linked to worse overall and cancer-specific survival, emphasizing the clinical importance of proactive surveillance strategies [11]. Age is also a critical risk factor, with the incidence of SPMs increasing among patients older than 70, although younger patients, as in this case, remain at risk [12]. In addition, diagnosis of NETs can also be challenging, such as for our patient, where a false negative liver biopsy conflicted with the PET-DOTATATE findings.

In our patient’s case, the original presentation of acute decompensated heart failure also raises the speculation for co-existing carcinoid heart disease. Although the patient had severe aortic and mitral regurgitation, carcinoid heart disease more commonly affects the right heart valves. Given the possibility of carcinoid heart disease, the excised aortic valve was examined and found to be inconsistent with valvulopathy induced by carcinoid disease. The severe valvulopathies that the patient presented with are more consistent with chronic mechanical injury secondary to a bicuspid valve instead of carcinoid heart disease, given the lack of plaque-like endocardial and valvular deposits characteristic of serotonin-induced injury.

The coexistence of celiac disease in our patient introduces another consideration. Celiac disease is strongly associated with small bowel lymphomas and adenocarcinomas, primarily due to chronic intestinal inflammation [13]. Although evidence directly linking celiac disease to gastroenteropancreatic NETs is limited, a recent report suggested that inflammation and hypergastrinemia may contribute to enterochromaffin-like cell carcinogenesis [14]. This association remains speculative but highlights the need for heightened awareness of overlapping risk factors in patients with multiple pathologies. Therefore, his presentation with nausea and vomiting can be attributed to both pancreatic NET and celiac disease, given the fact that his symptoms improved after starting treatment for both.

## Conclusions

In conclusion, this case underscores the diagnostic challenges in distinguishing between metastatic spread and a second primary malignancy in patients with NETs. Given the increased risk of SPMs in this population, clinicians should maintain a high level of vigilance when evaluating new lesions, even in sites atypical for metastasis. Early recognition and careful surveillance of potential SPMs may facilitate timely intervention and ultimately improve outcomes in this patient group. In our case, the patient likely had a common scenario of SPM despite there being the possibility of metastatic disease from his prior lung carcinoid tumor.
